# Patient Derived Xenografts Expand Human Primary Pancreatic Tumor Tissue Availability for *ex vivo* Irreversible Electroporation Testing

**DOI:** 10.3389/fonc.2020.00843

**Published:** 2020-05-22

**Authors:** Rebecca M. Brock, Natalie Beitel-White, Sheryl Coutermarsh-Ott, Douglas J. Grider, Melvin F. Lorenzo, Veronica M. Ringel-Scaia, Navid Manuchehrabadi, Robert C. G. Martin, Rafael V. Davalos, Irving C. Allen

**Affiliations:** ^1^Graduate Program in Translational Biology, Medicine and Health, Virginia Polytechnic Institute and State University, Roanoke, VA, United States; ^2^Department of Biomedical Engineering and Mechanics, Virginia Polytechnic Institute and State University, Blacksburg, VA, United States; ^3^Department of Electrical and Computer Engineering, Virginia Polytechnic Institute and State University, Blacksburg, VA, United States; ^4^Department of Biomedical Sciences and Pathobiology, Virginia-Maryland College of Veterinary Medicine, Blacksburg, VA, United States; ^5^Department of Basic Science Education, Virginia Tech Carilion School of Medicine, Virginia Polytechnic Institute and State University, Roanoke, VA, United States; ^6^Research and Development, AngioDynamics, Marlborough, MD, United States; ^7^Division of Surgical Oncology, Department of Surgery, School of Medicine, University of Louisville, Louisville, KY, United States

**Keywords:** irreversible electroporation, PDX, conductivity, inflammation, pancreatic cancer, ablation, IRE

## Abstract

New methods of tumor ablation have shown exciting efficacy in pre-clinical models but often demonstrate limited success in the clinic. Due to a lack of quality or quantity in primary malignant tissue specimens, therapeutic development and optimization studies are typically conducted on healthy tissue or cell-line derived rodent tumors that don't allow for high resolution modeling of mechanical, chemical, and biological properties. These surrogates do not accurately recapitulate many critical components of the tumor microenvironment that can impact *in situ* treatment success. Here, we propose utilizing patient-derived xenograft (PDX) models to propagate clinically relevant tumor specimens for the optimization and development of novel tumor ablation modalities. Specimens from three individual pancreatic ductal adenocarcinoma (PDAC) patients were utilized to generate PDX models. This process generated 15–18 tumors that were allowed to expand to 1.5 cm in diameter over the course of 50–70 days. The PDX tumors were morphologically and pathologically identical to primary tumor tissue. Likewise, the PDX tumors were also found to be physiologically superior to other *in vitro* and *ex vivo* models based on immortalized cell lines. We utilized the PDX tumors to refine and optimize irreversible electroporation (IRE) treatment parameters. IRE, a novel, non-thermal tumor ablation modality, is being evaluated in a diverse range of cancer clinical trials including pancreatic cancer. The PDX tumors were compared against either Pan02 mouse derived tumors or resected tissue from human PDAC patients. The PDX tumors demonstrated similar changes in electrical conductivity and Joule heating following IRE treatment. Computational modeling revealed a high similarity in the predicted ablation size of the PDX tumors that closely correlate with the data generated with the primary human pancreatic tumor tissue. Gene expression analysis revealed that IRE treatment resulted in an increase in biological pathway signaling associated with interferon gamma signaling, necrosis and mitochondria dysfunction, suggesting potential co-therapy targets. Together, these findings highlight the utility of the PDX system in tumor ablation modeling for IRE and increasing clinical application efficacy. It is also feasible that the use of PDX models will significantly benefit other ablation modality testing beyond IRE.

## Introduction

While prevention and early diagnosis are key to reducing cancer-related mortality, lack of treatments for many types of cancers, such as pancreatic cancer, has led to a stagnation in patient survival rates. New treatment options are vital to increasing the survival of these patients. Current progress in ablation modalities has shown success in clinical trials by improving patient morbidity and mortality, crossing barriers impassable for surgery and chemotherapy. However, the treatment parameters for these ablation modalities often derive from modeling data generated from *in vitro* or *ex vivo* studies using the mechanical or electrical properties of healthy tissue or cell line data from rodents. With only 15% of pancreatic cancer patients eligible for surgical resection, the amount of direct human tumor tissue available for testing is severely limited (1). Additionally, tumor tissue integrity declines over time once excised, leading to degradation of tissue mechanical and electrical properties that influence the accuracy of the *in vitro* and *ex vivo* modeling results compared to clinical application (2).

Beyond human applications, tumor ablation is also an emerging therapeutic strategy in the veterinary clinic, where canine and other large animal patients are often used in comparative oncology studies. While this offers several advantages in terms of access to sufficient malignant animal tissues from spontaneous tumors for analysis and modeling, these studies are often limited due to cost and a general paucity of validated reagents available to assess biological responses to treatment (3). Therefore, databases for tissue properties are often used (4, 5). However, this limits modeling for newer modalities and databases, in general, have been generated using healthy rather than malignant tissues, which can further complicate modeling accuracy (6). Immortalized cancer cell lines can also be utilized but are highly homogeneous and lack the secondary structures and biological complexity of the *in situ* tumor, resulting in significant deviations between the models and clinical observations (7).

**Figure 1:**
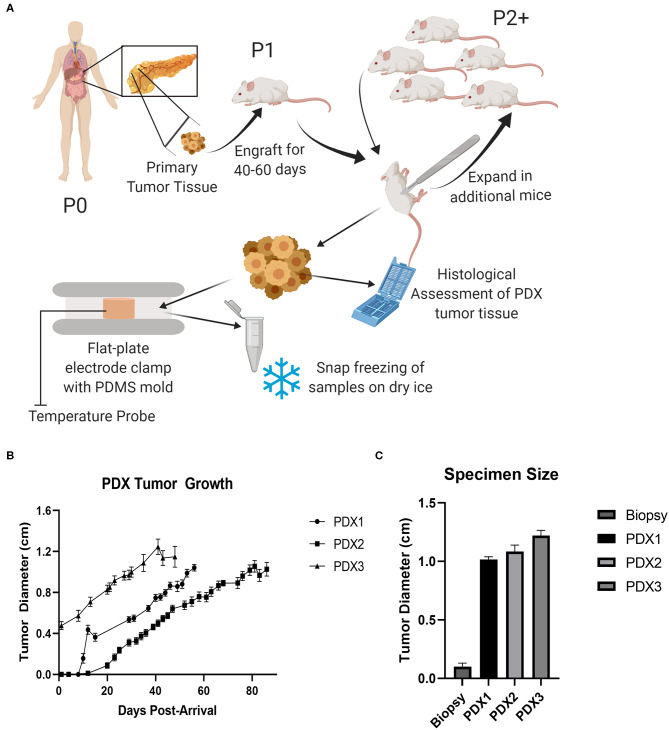
PDX models expand small tumor specimens for ablation testing. **(A)** Schematic of pancreatic cancer patient-derived xenograft model. Primary human pancreatic tumor tissue was implanted into an NSG (Passage 1) and allowed to progress, excised, and expanded into larger cohort of mice (Passage 2), and then collected for histological assessment and *ex vivo* testing. **(B)** Tumor growth curve from PDX model mice. SEM, *n* = 16–18 mice for each model. **(C)** Average maximum specimen size, SEM, *n* = 13–16.

To combat these limitations, we propose incorporating patient-derived xenograft (PDX) models to evaluate tumor ablation efficacy. PDX rodent models involve the engraftment of cancerous tissue from patients into immunocompromised animals, typically NOD *scid* gamma (NSG) mice. Over time, a small cancer biopsy will proliferate into a tumor that closely matches the biological complexity of the original patient's tumor. This tumor can then be excised and sub-cultured into exponentially greater numbers of mice to further propagate the tumor (Figure 1A). This process enables robust, high power modeling that is not possible utilizing direct from patient human specimens. While not yet widely utilized in the biomedical device development, PDX models have proven to be highly valuable tools in the pharmaceutical industry to determine individual patient responses to newly developed drugs (8). Thus, we foresee similar applications for the development of tumor ablation modalities. For the purpose of *ex vivo* tissue characterization and experimentation, the use of a flank PDX model as described here may be more desirable than an orthotopic model. While orthotopic methods, such as cell line injection models or genetic predisposition models like KPC, may lead to higher structured tumors, the amount of available tissue for testing can remain relatively small due to the size limitations *in situ* (9). There is also an increased morbidity risk to the host due to metastatic lesions (9). A flank model also allows for easier tumor size and progression assessments without the need for medical imaging equipment.

Here, we utilized PDX models of pancreatic cancer to evaluate tissue properties and ablation volumes following treatment with irreversible electroporation (IRE), a non-thermal electrical ablation modality that has shown significant improvements in Stage III and Stage IV pancreatic cancer patient survival in recent and ongoing clinical trials (10–13). Previous IRE modeling studies in the pancreas have been hindered by lack of surgical candidates and minimal tissue size, limiting data points for computational modeling of electrical properties and ablation volumes and statistical power in studies. These same limitation can be seen in several other ablation technologies, such as microwave ablation, which also relies on dielectric properties and the use of such databases for treatment planning (4, 6). Our results show that the PDX models retain the physiological and biological characteristics of the original patients' tumors. Likewise, the increased volume and quality of tumor specimens significantly improved the accuracy of ablation modeling. Further mechanistic studies were also possible, revealing several hallmarks of pancreatic cancer that were significantly impacted by IRE treatment. Together, these data support the integration of PDX models in tumor ablation studies and provide additional data to better define the mechanisms by which IRE treatment results in significantly prolonged survival in pancreatic cancer patients.

## Methods and Materials

### Experimental Animals

All experiments were conducted under institutional IACUC approval and in accordance with the NIH Guide for the Care and Use of Laboratory Animals. Murine Pan02 cells (NCI) were cultured with RPMI 1640 (ATCC) supplemented with 10% FBS (Atlanta Biologicals). Female NSG and C57Bl/6J mice were anesthetized and injected subcutaneously in right flank with 6 × 10^6^ cells in 100 μL of Matrigel (Corning, *n* = 5). Female NSGs engrafted with patient derived pancreatic cancer were generated by The Jackson Laboratory (detailed in Table 1). Mice were engrafted subcutaneously in right flank. Mice cohorts were confirmed by Jackson to have palpable masses before shipping to our facility and were received carrying passage 2 tumors (*n* = 16–18 mice per patient). Therefore, variance on beginning tumor size is expected between cohorts. All NSG mice were housed under immunocompromised conditions with autoclaved cages and water and irradiated chow. All mice were housed in SPF conditions with *ad lib* chow. Mice were monitored three times weekly until experimental endpoints were reached, tumors reached 1–1.6 cm in diameter calculated by the square root of the product of cross diameter measurements, or if considered clinically moribund.

**Table 1:** Human patient characteristics.

**Details**	**PDX specimens**			**Primary human specimen**				
Patient	1	2	3	A	B	C	D	E	F
Model id	J000077960	J000096053	TM01212	n/a	n/a	n/a	n/a	n/a	n/a
Primary site	Pancreas	Pancreas	Pancreas	Pancreas	Pancreas	Pancreas	Pancreas	Pancreas	Pancreas
Diagnosis	PDAC	PDAC	PDAC	Neuro-endocrine	PDAC	PDAC	PDAC	PDAC	PDAC
Tumor site	Pancreas	Lung	Pancreas	Pancreas	Pancreas	Pancreas	Pancreas	Pancreas	Pancreas
Tumor type	Primary	Metastatic	Primary	Primary	Primary	Primary	Primary	Primary	Primary
AJCC stage/grade	IV/2	IV/2	Unspecified	Low-Grade NET, 5 cm	IIb	III	IIa	III	III
Sex	F	M	M	M	F	F	F	F	F
Treatment	Naive	Naive	Naive	Chemo	Chemo	Chemo/XRT	Naïve	Chemo	Chemo
Age	68	64	Unspecified	52	67	77	72	49	63

*Human patient specimen characteristics used in the PDX models and primary human patient specimen characteristics for comparison tissues used in ex vivo assessments*.

### PDX Tissue Collection

Mice were euthanized according to IACUC protocol by carbon dioxide fixation followed by cervical dislocation. Tumor tissue was harvested post-euthanasia. A thin (2 mm) slice was taken laterally for histological assessment and remaining tissue stored in phosphate-buffered saline (PBS) during transport. All tissue was collected in groups of 3–5 mice and used for *ex vivo* testing within 2 h from excision to maintain tissue integrity.

### Human Patient Specimen Collection

This research study was approved by the Institutional Review Board (IRB) at the University of Louisville (02.0496). All potential pancreatic cancer patients undergoing either *in situ* IRE or pancreatectomy were asked for voluntary research participation from January 2016 to July 2018. A total of 6 pancreatectomy patients consented and were enrolled in this prospective study (detailed in Table 1). Research participation did not affect the treatment options of patients or inpatient care. All the participating patients were well-informed that they could withdraw their consent at any time during the study without affecting their treatment and ongoing care. The human patient data included in this manuscript is a subgroup of a larger cohort shown in a prior conference paper (14).

### IRE *ex vivo* Application and Tissue Properties

Fresh tumor tissue was cut into 2–3 cylindrical sections and placed into a polydimethylsiloxane (PDMS) mold to retain a cylindrical shape factor [thickness (t) = 0.56 cm, diameter = 0.6 cm]. A cylindrical shape ensures a known shape factor enabling simple calculation of the electrical conductivity with the following equation:

$$\begin{array}{l} \sigma =\frac{\left(I\cdot t\right)}{\left(V\cdot A_{c}\right)} \end{array}$$

where I is induced current, V is applied voltage, and A_c_ is cross-sectional area. The tissue-containing mold was placed between stainless steel, parallel-plate electrodes (Harvard Apparatus) connected to a BTX pulse generator (Harvard Apparatus).

A fiber optic probe (Luxtron m3300, LumaSense) was inserted to measure temperature at a frequency of 2 Hz during treatment. Parallel-plate electrodes ensure a uniform electric field is applied across the tissue sample. Prior to IRE pulsing, a 25 V, 100 μs pre-pulse was delivered in order to establish initial conductivity. A total of 100 IRE pulses were applied to the sample, with 100 μs pulse width and electric fields between 0 and 3000 V/cm. Changes in conductivity during electroporation were assumed to take place primarily during the first pulse. Thus, the average current value recorded during the last 5 μs of the first IRE pulse was used to calculate a single conductivity value for each sample.

### Numerical Investigation of Tissue Conductivity Response

A numerical model was constructed in COMSOL Multiphysics v5.4 (COMSOL Inc., Burlington, Massachusetts) to approximate the electric field distribution prior to (static) and during IRE (PDX1 and primary) in a two-needle electrode configuration (1.5 cm spacing/exposure, 100 pulses and 100 μs duration). Electric potential boundary conditions were set to maintain a voltage-to-distance ratio of 1,750 V/cm with all remaining external boundaries assigned as electrically insulating. The dynamic response to IRE was incorporated by applying the electrical conductivity curves to a tissue domain of dimension 10 × 8 × 8 cm. A “finer” mesh setting was selected and resulted in a mesh with 134,057 tetrahedral elements. The IRE ablation was estimated to occur at electric field values >500 V/cm, which is a previously reported threshold determined *in vitro* from primary murine pancreatic cancer cells (15). Associated Joule heating and thermal dissipation effects were modeled using a modified Bioheat equation and Joule heating term. A more detailed methodology can be found in previous work (16, 17).

### Histopathology

Tumor tissue sections were fixed in 10% formalin for at least 24 h, embedded in paraffin, and mounted on slides in 5 μm sections. Slides were stained with hematoxylin and eosin. Primary human patient specimen histopathology photomicrographs were provided by D.J.G from Virginia Tech Carilion School of Medicine. Histopathology analysis of all tissues was evaluated by a board-certified veterinary pathologist (S.C.O.).

### Gene Expression Evaluation

Tissue specimens were collected and snap frozen within 15 min post-treatment *ex vivo* and after 24 h *in vivo*. RNA was extracted from each sample via AllPrep DNA/RNA/Protein kit (Qiagen) and quantified via Nanodrop (Thermofisher). RNA was pooled equally for each electric field magnitude with 3–5 samples per treatment for a total of 540 ug RNA and converted into cDNA via RT_2_ First Strand (Qiagen). cDNA was plated on RT_2_ Profiler Human Cell Death Pathway, Human Cancer Pathway Finder, Mouse Innate and Adaptive Immunity, and Mouse Cancer Pathway Finder arrays (Qiagen) and run on ABI 7500 Fast Block (Thermofisher). RT_2_ Profiler plate results were normalized to individual untreated tumor tissues and plate housekeepers. ΔΔCT and fold regulation calculated via Qiagen Data Analysis Center. Gene expression data was analyzed by Ingenuity Pathway Analysis (Qiagen) and compared between individual patients and treatment dose. Examples of assay/program generated gene groupings are shown in Supplemental Figure 1. Heatmaps illustrating gene expression were generated using the web-based Heatmapper platform.

### Statistical Analysis

A Student's two-tailed *t*-test was utilized for comparisons between two experimental groups. Multiple comparisons were conducted using one-way and two-way ANOVA where appropriate followed by Mann-Whitney or Tukey post-test for multiple pairwise examinations. Statistical significance was defined as *p* < 0.05. All data are represented as the mean ± SEM.

## Results

### PDX Tumors Are Superior Models and Faithfully Recapitulate the *in situ* Tumor Microenvironment Compared to Cell Line Based Models of Pancreatic Cancer

To evaluate the potential of the PDX model to function as a surrogate for human *ex vivo* pancreatic cancer tissue in tumor ablation modality studies, we utilized xenografts from three separate human patients (Table 1). All patients were diagnosed with pancreatic ductal adenocarcinoma (PDAC) and tissue was collected from either the primary tumor mass (Patients 1 and 3) or a metastatic lung lesion (Patient 2). Xenografts were passaged (P2) in a total of 16–18 mice for each patient (Figure 1A). Each tumor was allowed to progress to at least 1 cm in diameter for each mouse (Figure 1B). The size of PDX derived tumor tissue available for downstream applications is significantly increased (1.11 ± 0.04 cm in diameter) in comparison to 16G human tissue specimen biopsies (0.1 ± 0.03 cm in diameter) that the original engraftment consisted of (Figure 1C). In addition to size advantages, the PDX tumor specimens were immediately available for downstream assessments following mouse necropsy, compared to typical delays in the range of hours for the direct from patient human specimens. Previous studies have shown significant changes in tissue electrical properties that occur by around one hour post-harvest (2). Thus, this immediate availability is a critical advantage of the PDX model for assessments of irreversible electroporation (IRE). Together, the increased size and quality of PDX specimens allowed for more robust testing of IRE treatment parameters and improved accuracy in electric property modeling.

**Figure 2:**
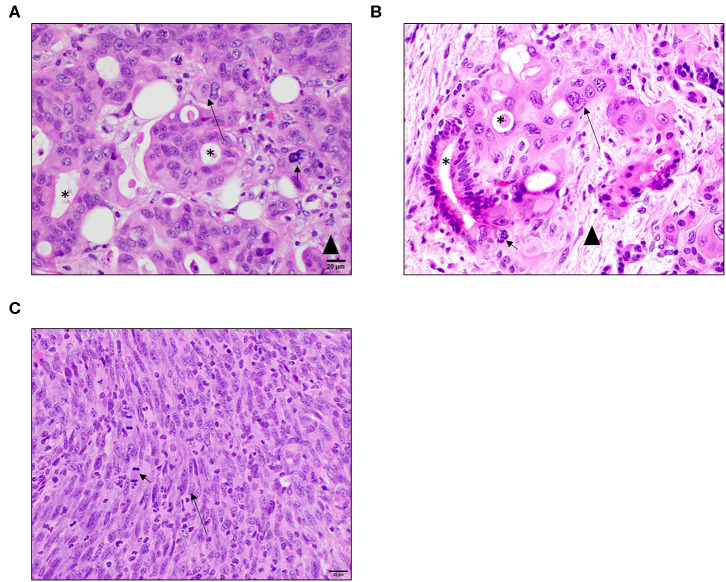
PDX tumors better recapitulate human PDAC histopathology and complexity compared to cell line based Pan02 models. **(A)** Representative image of PDX pancreatic tumor taken from Patient 1. Tumors from the PDX model exhibit similar characteristics as human patient samples including the formation of ductular structures and a similar tumor cell morphology. They have an identifiable collagenous stroma but not as robust as in patient samples. Mitotic figures are often abnormal. **(B)** Representative primary human PDAC tumor. Tumor cells from human patients often form ductular structures with lumens. Moderate to large amounts of fibrous connective tissue stroma separates tumor cells. Individual tumor cells are irregularly round with abundant amounts of eosinophilic cytoplasm and irregularly round nuclei. Mitotic figures are often abnormal. **(C)** Representative image of a Pano2 tumor expanded in a NSG mouse. Tumors derived from Pan02 cells lack the formation of ducts. Tumor cells are arranged in vague streams. They are more spindle in shape with less cytoplasm and elongated nuclei. The tumor stroma is scant. Mitotic figures are of normal morphology. Short arrows indicate mitotic figures, asterisks indicate ductal structures, and long arrows indicate elongated nuclei, and arrowhead indicates tumor stroma tissue. All images are HE stain and were taken at 40x magnification.

To evaluate the histopathological features of the P2 tumors generated in the PDX model, specimens were collected at necropsy, fixed, and processed for hematoxylin and eosin (H&E) staining. These specimens were compared to the following: (1) the P0 donor histological reports provided with each PDX mouse; (2) PDAC pathology from reference sources; (3) PDAC pathology comparisons with specimens directly from human patients; and (4) Cell line (Pan02) derived tumor tissue from NSG mouse flank injections. Specimens were evaluated by either a board-certified veterinary (S.C.O.) or human (D.J.G.) pathologist. The analysis revealed that the PDAC tumors, in general, faithfully recapitulated the common histopathologic features and biological complexity of the patient's original tumors and were highly consistent with PDAC pathology. PDX tumors exhibit irregularly round cells that often form prominent ductular structures with lumens containing necrotic debris, sloughed cells, or small amounts of mucinous secretion (Figure 2A). Neoplastic cells exhibit significant differences in cell size and shape across the tumor cell population which is consistent with malignancy (Figure 2A). Individual tumor cells have abundant eosinophilic cytoplasm (Figure 2A). An identifiable but not prominent fibrovascular tumor stroma is also present (Figure 2A). All of these features were also readily observed in specimens collected from the original donors (available from the Mouse Tumor Biology Database) and primary human PDAC tumor samples (Figure 2B). However, in the Pan02 tumors, neoplastic cells are elongated and spindle-shaped with no evidence of glandular formation and exhibit fewer cytological criteria of malignancy with uniform size and shape (Figure 2C). They form vague streams with minimal fibrous connective tissue stroma. Mitotic figures are prominent but are not as bizarre as in PDX model and human specimens (Figure 2C). Individual cells contain significantly less cytoplasm than PDX tumors and are more densely packed (Figure 2C). Thus, based on histopathology, these data indicate that the PDX tumors faithfully recapitulate the human pancreatic tumor microenvironment and are more physiologically accurate compared to cell line derived Pan02 tumor models.

### Irreversible Electroporation (IRE) Electric Field Distributions Are Consistent Between PDX and Human Tissue Specimens

**Figure 3:**
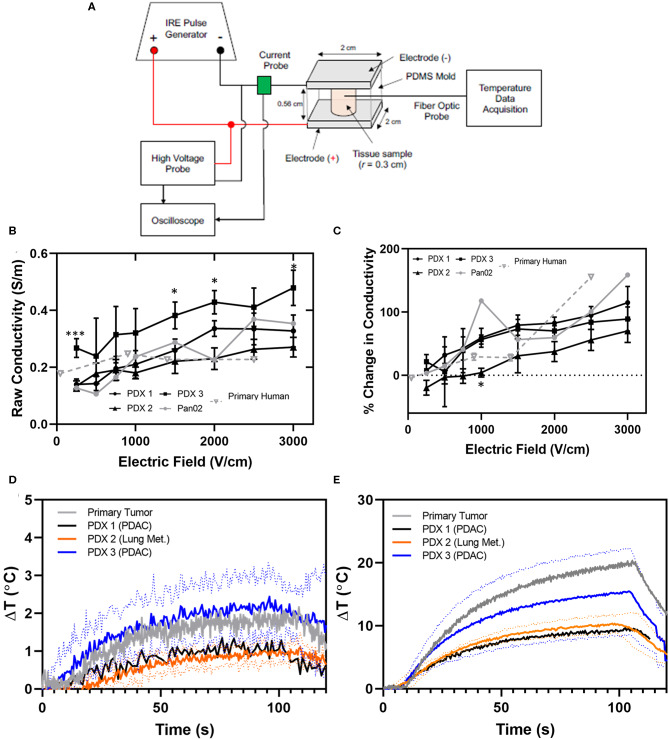
PDX and primary human tissues exhibit similar conductivity and temperature changes during IRE application. **(A)** Schematic of IRE treatment and tissue characteristic assessment *ex vivo*. **(B)** Raw and **(C)** percent change in conductivity for PDX (*n* = 3–6), primary human tissues (*n* = 2–3), and Pan02 tumor tissue (*n* = 1) for each electric field were collected at electric field magnitudes ranging from 0 to 3000 V/cm during IRE application. Conductivity values were calculated based on the average current recorded during the last 5 μs of the first IRE pulse. SEM, 2-way ANOVA (Pan02 not considered due to low *n*-value), *p*-value * < 0.05, *** < 0.001. Change in temperature induced by IRE at **(D)** 1,000 V/cm and **(E)** 2,500 V/cm were recorded throughout pulse application.

In an effort to improve computational modeling and refine patient treatment algorithms utilized in IRE treatment, we utilized specimens from the PDX tumors to evaluate and refine electric field distribution simulations. The overwhelming majority of tumor ablation modalities, including IRE, have been developed and modeled utilizing either healthy tissue or specimens collected from cell line derived tumors. Moreover, these collections have been without measuring the effect of IRE itself on tissue conductivity, utilizing a “static” model that does not incorporate dynamic conductivity changes over treatment time. Based on the findings from the histopathology assessments, we hypothesized that PDX tumors would provide a more accurate model system that better recapitulates the electrical properties of patient's tumors both for initial dielectric properties and dynamic changes. To evaluate this hypothesis, PDX tumors from 3 individual patients, and primary human pancreatic tumor tissue from 6 individual patients collected during surgery (Table 1) (14) were subjected to *ex vivo* IRE applications utilizing parallel-plate electrodes and compared (Figure 3A). Individual tumors from PDX patients were observed to have different raw conductivities, with PDX3 differing significantly from primary human tissues at several electric fields tested (Figure 3B). However, when evaluated as a percent change in conductivity by considering the different initial base-line conductivities of the tissue prior to IRE application, these differences in conductivity were reduced between the 3 PDX patients to closer to that of the primary tissues for most electric fields tested, although PDX tumors from patient 1 and 3 exhibited higher raw conductivity overall than PDX patient 2 (Figures 3B,C). Due to the low number of replicates it is difficult to determine whether the discrepancy is due to true tissue differences or experimental error. As expected, only minimal changes in temperature were observed in all tissues at conditions below 1,000 V/cm, with Joule heating more severe at higher electric field magnitudes (Figures 3D,E).

**Figure 4:**
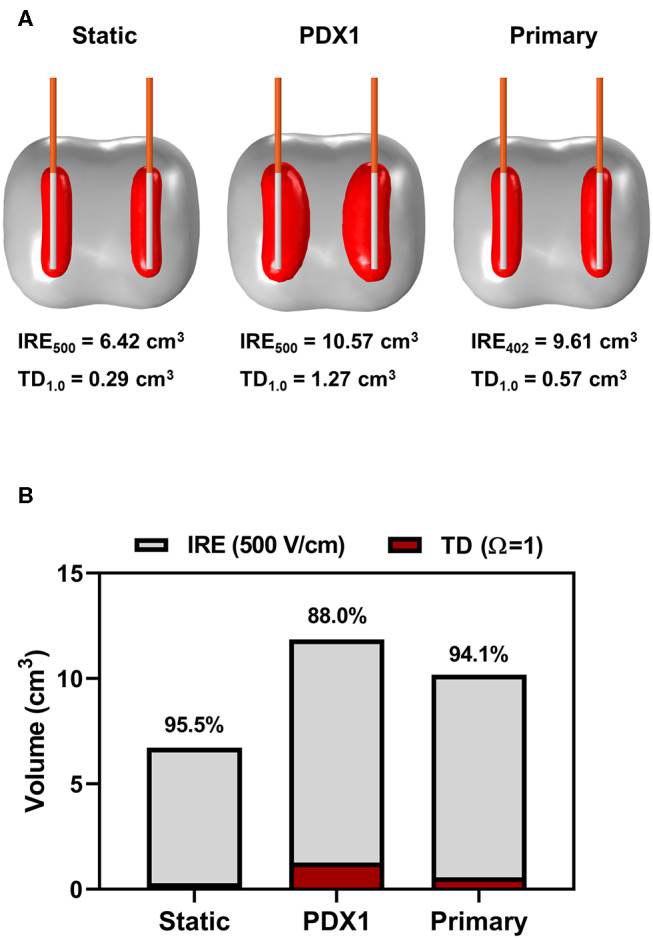
Modeling of PDX and primary human tissues results in similar predicted ablation sizes and damage contributions. **(A)** COMSOL model depiction of the predicted ablation area at 500 V/cm (gray) and thermal damage area at Ω = 1.0 (red) resulting from 100 pulses, 100 μs on time, and 1,750 V/cm voltage to distance ratio modeling clinical IRE. **(B)** Quantification of COMSOL model of predicted contributions of IRE and thermal damage to tumor ablation model.

The PDX electrical conductivity data were utilized in COMSOL simulations and modeling to predict ablation sizes and tissue damage contributions. Analysis of IRE with a two monopolar configuration (1.5 cm spacing/exposure, 1,750 V/cm, 100 pulses and 100 μs duration) determined that electric field distributions did not significantly differ between the PDX tumors and primary tumor tissues from patients (Figure 4). For example, using the PDX tumor from patient 1 (PDX-1) as a representative tumor, there is a large discrepancy in predicted ablation volume and geometry between the static case and incorporating dynamic conductivity measured from PDX-1 and primary tumor tissue (Figure 4A). Thermal damage volumes were obtained by applying the Arrhenius equation as described previously (18). Using an IRE threshold of 500 V/cm and a thermal damage threshold of Ω = 1.0, the total ablation areas for static case, PDX-1, and primary tumor consisted of non-thermal IRE volumes accounting for 95.5, 88.0, and 94.1% of the total ablation volume (Figure 4B). The thermal damage accounted for 4.5, 12, and 5.9% of the total ablation volume, respectively (Figure 4B). These data have direct translational implications and suggest that further optimization of our treatment parameters (such as lower on-time [90 μs], lower voltage, or thermal mitigation strategies) are possible and could decrease the potential for thermal damage (19).

### IRE Treatment Impacts Critical Hallmarks of Pancreatic Cancer

**Figure 5:**
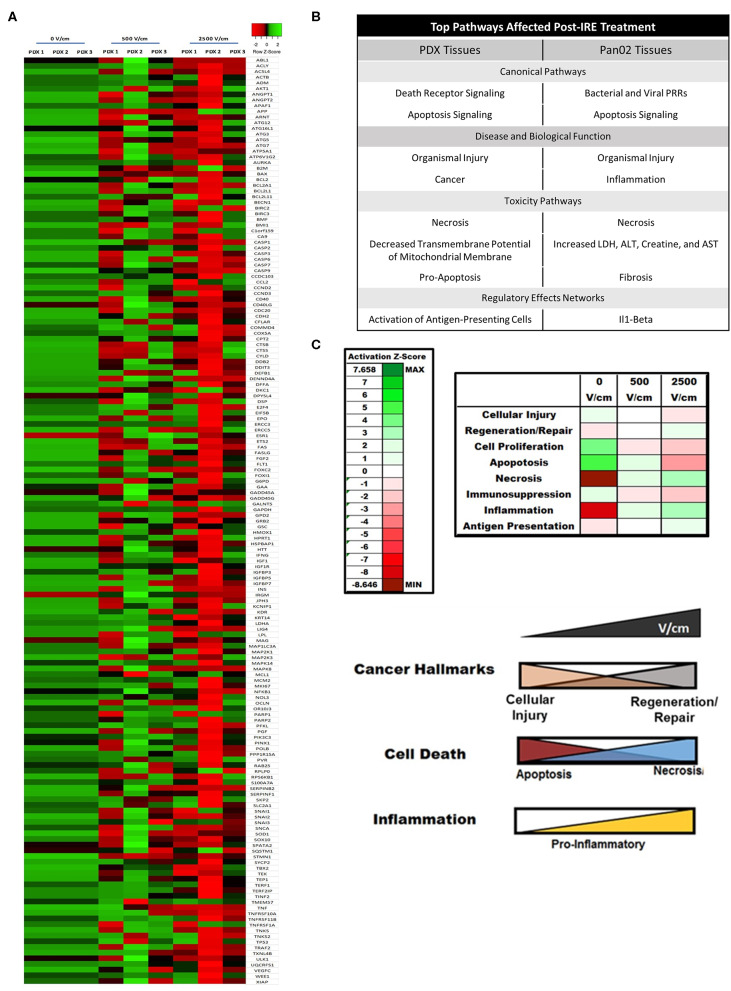
IRE induces patient- and dose-dependent gene expression changes in PDX pancreatic tumors. **(A)** Gene expression arrays were utilized to evaluate changes in the expression of genes associated with cancer and cell death following IRE treatments at 0, 500, and 2,500 V/cm. A heatmap of the expression data was generated (z-score ranking ±3). *N* = 3–6 specimens in each group. **(B)** Summary table of dominate biological pathways affected by IRE in pancreatic tumor tissue from human PDX samples and from murine Pan02 samples. **(C)** IRE significantly alters cancer hallmark and immunosuppressive biological pathways in PDX pancreatic tumor models. IPA analysis of affected biological pathways assigned Z-scores based on predicted impact from individual gene expression changes. 0, 500, and 2500V/cm IRE treated tissues were compared and showed significantly increased necrosis, regeneration/repair, and inflammation signaling. Diagram of dose-dependent effect of IRE on biological pathways involved in cancer, cell death, and inflammation.

To complement the electrical property assessments, we also utilized the PDX models to identify biological signaling networks associated with pancreatic cancer that are impacted by IRE treatment. Gene expression profiling was utilized to identify genes dysregulated by treatment and Ingenuity Pathway Analysis (IPA) utilized these data to predict the biological functions significantly impacted by IRE as previously described (20–22). We identified 150 individual genes associated with cancer hallmarks and cell death were predominantly up-regulated in untreated PDX tumor specimens (Figure 5A). Indeed, we observed similar expression patterns for these genes in all three PDX specimens (Figure 5A). Following IRE treatment with 500 V/cm, we observed a general downregulation in in these the majority of these genes; however, there was a wide disparity between the individual genes down-regulated per patient. Patient 1 had the greatest number of genes significantly downregulated (80/150; 53%), followed by Patient 3 (45/150; 30%). Patient 2 also had a significant number of genes down-regulated following IRE treatment (29/150; 19.3%). However, these were significantly less compared to the PDX tumors from Patient's 1 and 3. Clustering analysis revealed significant similarities in gene transcription patterns in treated PDX tumors from Patient's 1 and 3, whereas the treated tumors from Patient 2 clustered separately (Figure 5A). This observation is potentially due to the tumors from Patient's 1 and 3 being primary PDAC, compared to the metastatic lung PDAC tumor from Patient 2 (Table 1). This could indicate differences in the biological responses to IRE between primary and metastatic tumors at lower V/cm. At the higher 2,500 V/cm, we observed and even greater down-regulation in individual gene expression associated from all 3 PDX patient specimens. As with the 500 V/cm specimens from Patient 1 and 3 clustered together, while the specimen from Patient 2 clustered separately (Figure 5A). However, at the higher V/cm, the differences were due to an increased number of genes significantly down regulated in the PDX tumors from patient 2 (132/150; 88%), compared to the number down regulated from Patient 1 (88/150; 58.7%) and Patient 3 (74/150; 49.3%) (Figure 5A). Pan02 tumors treated at 2,000 V/cm show similar expression trends in downregulation as those seen at 2,500 V/cm in the PDX tissues between related genes listed in Supplemental Figure 2.

IPA analysis of the gene expression data identified 8 pathways significantly dysregulated in the pancreatic cancer PDX tumors, as well as, murine Pan02 tumors following IRE treatment (Figure 5B). These pathways were grouped as either canonical pathways, disease and biological function pathways, toxicity pathways, or regulator effects networks and included the following: death receptor signaling; apoptosis signaling; organismal injury, cancer, necrosis, decreased transmembrane potential of mitochondrial membrane, pro-apoptosis, and activation of antigen presenting cells (Figure 5B). Many of these pathways were consistent between PDX and Pan02 groups with several overlapping, such as necrosis and organismal injury. Several of these pathways were identified as having the largest change in global gene expression from baseline (0 V/cm) to maximum treatment (2,500 V/cm) and could be grouped even further into 3 functional categories: cancer hallmarks, cell death, and inflammation (Figure 5C). Intriguingly, we identified several counterbalancing trends in gene expression patterns for each category. For cancer hallmarks, cellular injury and regeneration/repair were the dominant functions impacted by treatment. At baseline, there was increased cellular injury signaling in the PDX tumors, which dose dependently declined following IRE treatment (Figure 5C). Conversely, we observed an increase in regeneration and repair signaling with increased IRE dosage (Figure 5C). Similar data was observed for cell death where apoptosis signaling was increased at baseline and decreased with higher dosages of IRE; whereas, necrosis signaling was lower at baseline and increased with higher dosages of IRE (Figure 5C). Pancreatic tumors are typically immunosuppressive (Figure 5C) (1); however, following IRE, we observed a dose-dependent increase in pro-inflammatory inflammation signaling and antigen presentation potential in the PDX specimens. IPA analysis also identified several general biology signaling pathways that were significantly impacted by IRE treatment, including decreased transmembrane potential of mitochondria (Figure 5B). While we did not observe a dose dependent trend in this pathway, its finding is intriguing and is consistent with reduced tumor viability following IRE treatment.

**Figure 6:**
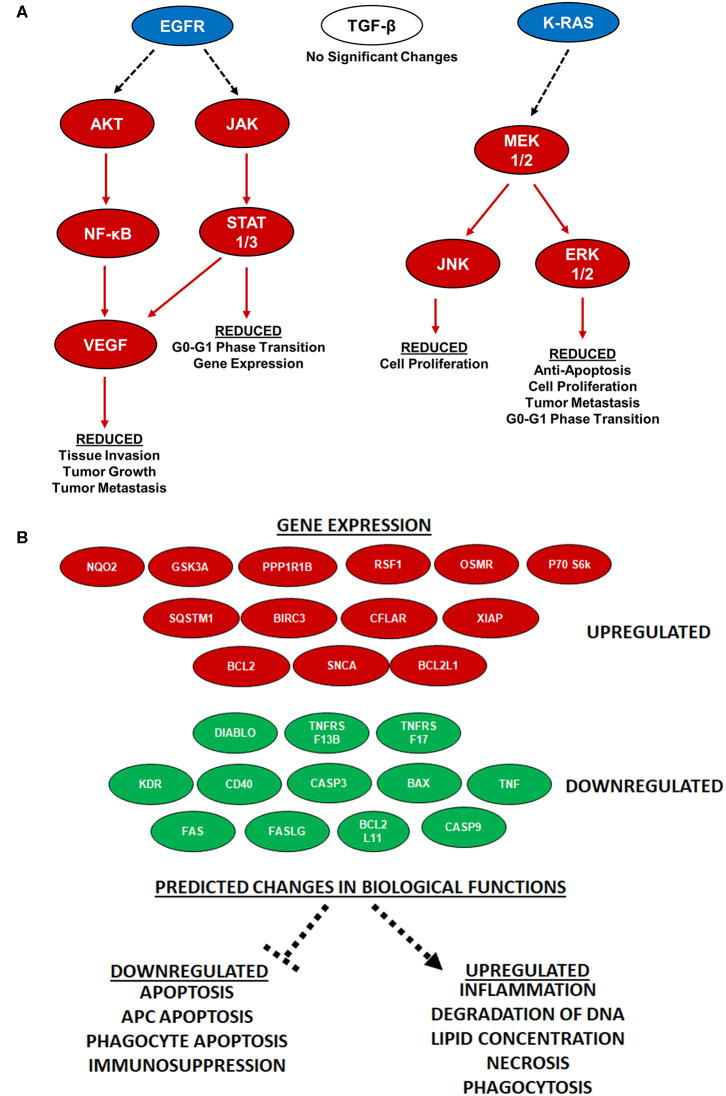
IRE treatment potently attenuated KRAS and EGFR signaling and increases antigen presentation. **(A)** Specific pancreatic cancer-related pathways were analyzed via IPA and showed significant alternations in gene expression of KRAS and EGFR pathway signaling molecules following IRE treatment at 2500 V/cm. *n* = 3–6 specimens in each group. **(B)** Antigen presentation pathways are significantly increased in PDX tumors following IRE treatment. Twenty-five genes were identified as being key regulators associated with the increase in antigen presentation signaling following IRE treatment (red is up-regulated; green is down-regulated). These genes are predicted to impact the function of 9 key drivers of antigen presentation and impact the biological functions shown at the bottom of the schematic, all predicted to result in increased antigen presentation.

While a diverse range of biological signaling mechanisms can be significantly dysregulated during pancreatic cancer, there are 12 distinct core signaling pathways found dysregulated in 60–100% of human clinical cases (23). We specifically evaluated these pathways in the PDX tumors to determine if any were specifically altered by IRE treatment at 2,500 V/cm. Our analysis revealed that most of these pathways were unaltered. However, IPA revealed that the changes in gene transcription identified in our core signaling analysis were most likely associated with decreased signaling downstream of EGFR and K-RAS (Figure 6A). Specifically, IPA revealed significant decreases in AKT, JAK, NF-κB, VEGF, and STAT1/3 signaling downstream of EGFR (Figure 6A). We also identified significant decreases in MEK1/2, JNK, and ERK1/2 signaling downstream of K-RAS (Figure 6A). Intriguingly, even though TGF-β signaling is also upstream of most of these signaling mechanisms and one of the core signaling pathways in pancreatic cancer, the gene expression data did not identify a significant impact of IRE treatment on TGF-β signaling (Figure 6A). Furthermore, IPA predicted that the downregulation of these specific pathways would result in the reduction of a variety of biological functions critical to pancreatic cancer progression (Figure 6A). For example, the reduced EGFR signaling pathways were predicted to result in reduced tissue invasion, tumor growth, tumor metastasis, G0-G1 phase transition, and gene expression (Figure 6A). Likewise, the reduced K-RAS signaling is predicted to result in reduced cell proliferation, anti-apoptosis signaling, cell proliferation, tumor metastasis, and G0-G1 phase transition (Figure 6A). The identification of specific pathways altered by IRE treatment provides insight regarding potential biomarkers to monitor for treatment progress or evaluate for treatment failure, patient selection criteria, and combination therapeutic strategies.

Several recent studies have revealed that IRE and other electroporation-based tumor ablation modalities significantly alters the tumor microenvironment and immune system activation (21, 24–28). In general, these studies show that irreversible electroporation results in a shift in the immunosuppressive tumor microenvironment to one that is more pro-inflammatory and anti-tumorigenic. Likewise, several of these studies reveal an increase in the systemic anti-tumor immune response and improved engagement of the adaptive immune system targeting metastatic cells (21, 26). However, these findings have yet to be applied to pancreatic cancer. Our IPA analysis revealed that IRE treatment induced gene expression patterns that were consistent with an up-regulation of antigen presentation in the PDX tumor specimens (Figure 5). This increase in antigen presentation was due to the significant up-regulation of 13 genes and down-regulation of 12 genes identified or predicted by the IPA analysis to be associated with this biological function (Figure 6B). Globally, the changes identified in these genes are predicted to significantly down-regulate apoptosis, antigen presenting cell apoptosis, phagocyte apoptosis, and immunosuppression (Figure 6B). Conversely, the changes in these genes are predicted to increase inflammation, degradation of DNA, lipid concentration, necrosis, and phagocytosis (Figure 6B). It is important to note that the PDX model is devoid of most immune system components. Thus, the changes shown reflect the direct effects of IRE on the tumor cells, without the confounding effects of the immune system.

## Discussion

Tumor specimens from human patients have the most clinical and physiological relevance for modeling tumor ablation technologies. However, the lack of high-quality and low-quantity patient tissue for robust modeling has impacted the field. Other methods, such as cell lines (i.e., immortalized cells maintained as monoculture colonies), are useful for understanding basic mechanistic insight related to cancer biology and mechanisms of tumor ablation. However, prolonged propagation and maintenance of these lines has led to the loss of many of the biological characteristics associated with the original tumor. Their behavior following treatment often does not recapitulate the responses observed in the patient tumor. Even cell line co-culture and organoid models have significant limitations. To circumvent this for IRE ablation, we utilized PDX derived tumors that faithfully recapitulates the morphological features of the patient's pancreatic cancer and is highly effective in generating abundant volumes of tumor specimens for electrical property and ablation modeling. Histopathological analysis shows similar morphological features in the PDX model tumors as compared to donor tissue that are of higher complexity than immortalized cell line-generated tumors. In terms of electrical conductivity, the PDX tumor data was fairly consistent with the available data from the primary human patient specimens, allowing for the development of a predictable model for clinical application. Thermal response differences in the PDX models were also minimal and may be due to tissue necrosis that has been known to occur in patient tumor tissue (29). This implies that PDX tissues are effective surrogates for human primary tissues in terms of electrical and thermal responses to electroporation pulses and potentially other targeted ablation modalities.

The PDX tumors evaluated in the current study included 2 primary PDAC specimens from the pancreas and 1 metastatic PDAC specimen from the lung. This allowed us to robustly evaluate 8 different electric fields (ranging from 0 to 3,000 V/cm) using 3–6 specimens for each parameter and patient. The resulting data were then utilized to improve the accuracy of our predicted ablation area and thermal damage area modeling. Together, these data will ultimately be incorporated into the IRE treatment planning algorithms to improve patient treatment outcomes in the clinic. While we did observe differences in the raw conductivity and change in temperature between patients, the percent changes were not statistically different between patient samples or tumor origin for most electric field magnitudes. We originally hypothesized that individual differences in the tumor microenvironment, genetic differences between patients, or differences in tissue derived from metastatic sites compared to primary tumors may have different physiological or electrical properties that could impact IRE treatment. However, based on the findings here for the specimens and treatment parameters evaluated, we did not observe any significant differences between individual patient's PDX tumors.

Gene expression analysis on the treated PDX tissues gives insight to the mechanism behind IRE's ablative ability. The biological pathways and functions between the 3 different PDX models were consistent prior to treatment and, in general, had similar changes post-treatment. Our analysis revealed a strong shift from apoptosis to necrosis following treatment, which is consistent with previous findings in pre-clinical mouse studies (21). As pancreatic tumors are typically classified as “cold” or non-immunogenic, a more pro-inflammatory type of cell death could lead to tumor microenvironmental changes that make it susceptible to co-immunotherapy options, such as checkpoint inhibitors (30). Clinically, pancreatic cancer has had a lack of response to most individually-applied immunotherapeutics (31). IRE may improve immunotherapy efficacy as it triggers a shift from an inherently immunosuppressive microenvironment to one that is more pro-inflammatory and subsequently anti-tumor (21, 30). Lastly, the impact of IRE on downstream KRAS and EGFR signaling could prove vital for determining treatment strategies. These pathways are commonly dysregulated in pancreatic cancer patients (1, 32, 33). Our data indicates that these pathways are highly down-regulated following IRE. This downregulation can alter several relevant biological functions for cancer biology, including proliferation, cell death, invasion, and metastasis. Interestingly, we did not observe significant changes in other pathways commonly associated with pancreatic cancer, such as TGF-β signaling, which is highly involved in pancreatic cancer pathophysiology (34). Thus, it is tempting to speculate that components of the KRAS and EGFR signaling pathway may prove to be therapeutic targets post-IRE or effective biomarkers to gauge treatment efficacy. Likewise, these data may suggest that pancreatic cancer patients with underlying mutations in genes associated with TGF-β signaling may have reduced responses to IRE based therapeutic strategies.

While PDX models have become an essential tool in drug discovery applications, these models have yet to be widely adopted in biomedical engineering and device development. The data presented here supports their further incorporation in these fields and demonstrates their utility in expanding malignant tissues that retain morphological and clinically relevant properties. Their use allows for the robust investigation of potential treatment associated biomarkers and co-therapy options. In the context of IRE, increased use of PDX models are anticipated to improve our understanding of tissue electrical properties in both primary and metastatic tumors, and these data will improve ablation zone predictions, ultimately leading to more precise and predictable clinical applications in the pancreas.

## Data Availability Statement

The datasets generated for this study are available on request to the corresponding author.

## Ethics Statement

The studies involving human participants were reviewed and approved by University of Louisville Institutional Review Board. The patients/participants provided their written informed consent to participate in this study. The animal study was reviewed and approved by Virginia Polytechnic Insitute and State University Insititutional Animal Care and Use Committee.

## Author Contributions

IA, RD, and NM contributed to the concept and design of the study. RB and VR-S monitored animals and collected animal tissues. RM provided primary human tissues. NB-W performed *ex vivo* IRE and tissue property assessments. SC-O and DG assessed histology and provided photomicrographs. ML provided COMSOL modeling. RB performed tissue processing and RNA evaluation. IA analyzed IPA results. NB-W, ML, SC-O, and IA wrote sections of the manuscript. RB wrote the original draft of the manuscript and editing.

## Conflict of Interest

IA, ML, NB-W, and RD are inventors on pending and issued patents related to the work. NM is employed by AngioDynamics. The authors declare that this study received funding from AngioDynamics. The funder had the following involvement in the study: study design. The remaining authors declare that the research was conducted in the absence of any commercial or financial relationships that could be construed as a potential conflict of interest.
