# Supplementary figures and images for: Patient Derived Xenografts Expand Human Primary Pancreatic Tumor Tissue Availability for ex vivo Irreversible Electroporation Testing
Source: Front Oncol. 2020 May 22;10:843. doi: 10.3389/fonc.2020.00843 (PMC7257557; doi:10.3389/fonc.2020.00843)

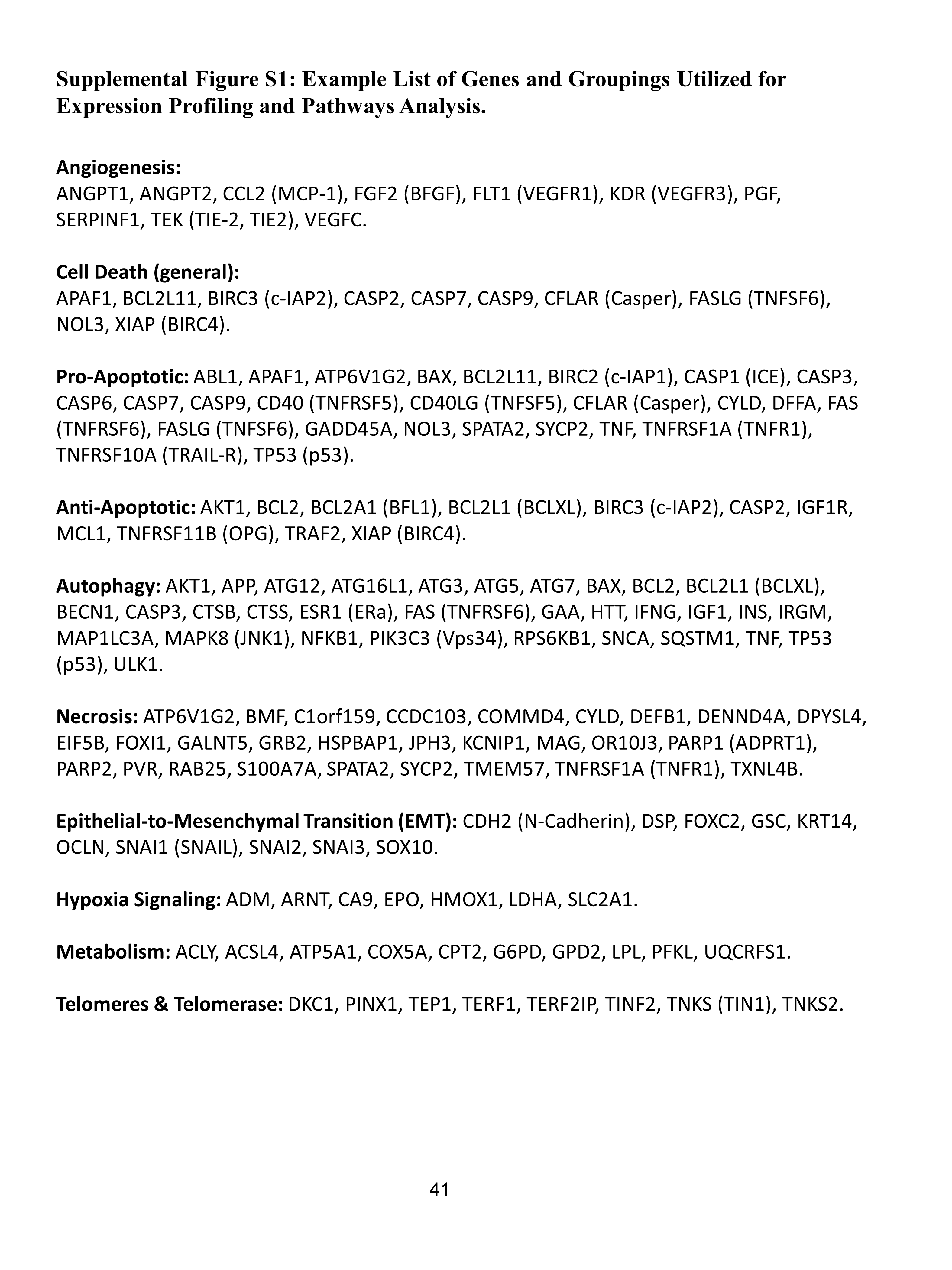

Supplement: Supplementary file 1 [file Image_1.TIF]

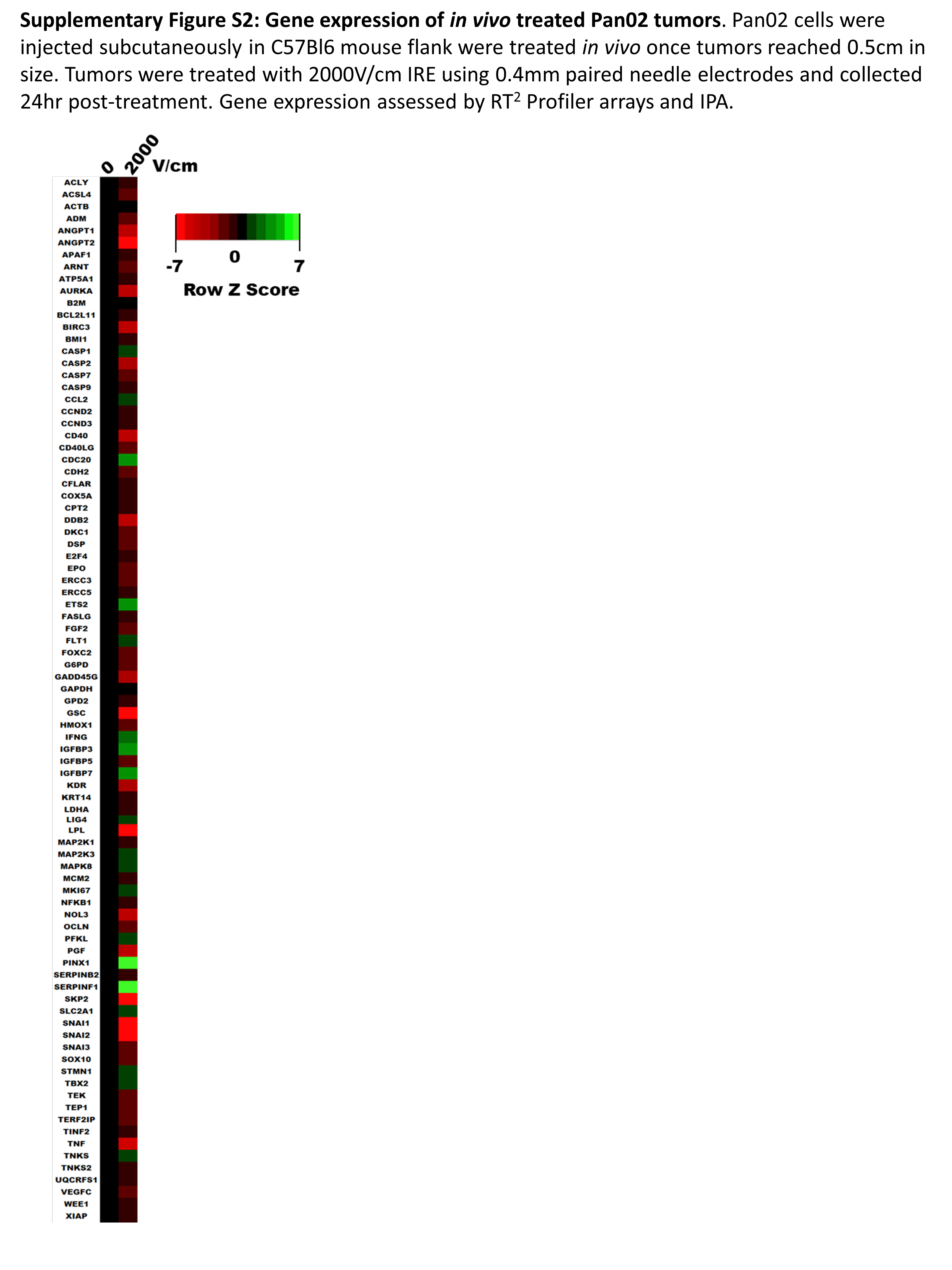

Supplement: Supplementary file 2 [file Image_2.TIF]
